# Associations of cerebral amyloid beta and tau with cognition from midlife

**DOI:** 10.1002/alz.14060

**Published:** 2024-07-22

**Authors:** Mitzi M. Gonzales, Adrienne O'Donnell, Saptaparni Ghosh, Emma Thibault, Jeremy Tanner, Claudia L. Satizabal, Charles S. Decarli, Georges El Fakhri, Keith A. Johnson, Alexa S. Beiser, Sudha Seshadri, Matthew Pase

**Affiliations:** ^1^ Department of Neurology Cedars Sinai Medical Center Los Angeles California USA; ^2^ Glenn Biggs Institute for Alzheimer's & Neurodegenerative Diseases University of Texas Health Science Center at San Antonio San Antonio Texas USA; ^3^ Department of Neurology University of Texas Health Science Center at San Antonio San Antonio Texas USA; ^4^ The Framingham Heart Study Framingham Massachusetts USA; ^5^ Department of Biostatistics Boston University School of Public Health Boston Massachusetts USA; ^6^ Department of Neurology Boston University Chobanian & Avedisian School of Medicine Boston Massachusetts USA; ^7^ Department of Radiology Massachusetts General Hospital and Harvard Medical School Boston Massachusetts USA; ^8^ Department of Neurology University of California Davis Sacramento California USA; ^9^ Department of Population Health Sciences University of Texas Health Science Center at San Antonio San Antonio Texas USA; ^10^ Center for Neuroscience University of California Davis Davis California USA; ^11^ Department of Radiology Yale School of Medicine New Haven United States; ^12^ Department of Neurology Massachusetts General Hospital and Harvard Medical School Boston Massachusetts USA; ^13^ Department of Neurology Brigham and Women's Hospital and Harvard Medical School Boston Massachusetts USA; ^14^ School of Psychological Sciences Turner Institute for Brain and Mental Health Monash University Clayton VIC Australia

**Keywords:** amyloid beta, cognition, midlife, PET imaging, tau

## Abstract

**INTRODUCTION:**

Understanding early neuropathological changes and their associations with cognition may aid dementia prevention. This study investigated associations of cerebral amyloid and tau positron emission tomography (PET) retention with cognition in a predominately middle‐aged community‐based cohort and examined factors that may modify these relationships.

**METHODS:**

^11^C‐Pittsburgh compound B amyloid and ^18^F‐flortaucipir tau PET imaging were performed. Associations of amyloid and tau PET with cognition were evaluated using linear regression. Interactions with age, apolipoprotein E (APOE) ε4 status, and education were examined.

**RESULTS:**

Amyloid and tau PET were not associated with cognition in the overall sample (*N* = 423; mean: 57 ± 10 years; 50% female). However, younger age (< 55 years) and APOE ε4 were significant effect modifiers, worsening cognition in the presence of higher amyloid and tau.

**DISCUSSION:**

Higher levels of Aβ and tau may have a pernicious effect on cognition among APOE ε4 carriers and younger adults, suggesting a potential role for targeted early interventions.

**Highlights:**

- Risk and resilience factors influenced cognitive vulnerability due to Aβ and tau.
- Higher fusiform tau associated with poorer visuospatial skills in younger adults.
- APOE ε4 interacted with Aβ and tau to worsen cognition across multiple domains.

## 1 BACKGROUND

Alzheimer's disease (AD) is biologically defined by the presence of amyloid beta (Aβ) plaques and hyperphosphorylated tau‐derived neurofibrillary tangles,^1^ which can be visualized in vivo using position emission tomography (PET) imaging.^2^ According to the Aβ cascade hypothesis,^3^ AD pathogenesis initiates decades before symptom onset with the accumulation of Aβ plaques.^4^ Aβ plaques appear to play a role in the propagation of tau outside the transenthorinal cortex, which is associated with cognitive impairment.^5^ In cognitively intact older adults with elevated Aβ, neocortical tau is a strong predictor of cognitive decline and is associated with elevated risk of conversion to mild cognitive impairment (MCI) and AD dementia.^6^, ^7^, ^8^ Some studies conducted in older adults have reported that entorhinal tau is associated with poorer memory performance and accelerated memory decline independent of Aβ,^9^, ^10^ whereas others have suggested that Aβ and tau interact to induce cognitive decline.^6^, ^7^

Given the long quiescent period between Aβ deposition and clinical diagnosis,^11^, ^12^ elevated Aβ levels have become a target for dementia prevention trials.^13^ Efforts are also underway to identify the earliest time point at which meaningful elevations in tau PET can be detected. Among individuals with an autosomal dominant mutation for AD, elevations in tau PET emerge proximally to symptom onset.^11^, ^14^ In contrast, Aβ PET positive older adults in the cognitively asymptomatic stage have been shown to display increased tau PET retention in the medial temporal, precuneus, posterior cingulate, and lateral parietal lobe relative to age‐matched Aβ negative individuals.^15^, ^16^, ^17^ Even among Aβ PET negative individuals, older age has been associated with higher mesial temporal lobe tau PET binding.^18^ In a cohort study of middle‐aged adults enriched for parental history of AD, concurrent Aβ and tau PET positivity were retrospectively associated with accelerated cognitive decline.^19^ However, it remains unknown if the full distribution of Aβ and tau PET retention are cross‐sectionally associated with cognition at midlife.

The primary aim of the current study was to evaluate the associations of the continuous range of Aβ and tau PET retention with cognition in a predominately middle‐aged subset of the Framingham Heart Study (FHS).^20^ Prior work has demonstrated variability in the rate of cognitive decline even among individuals with elevated Aβ and tau,^21^ highlighting the importance of determining both risk and resilience factors for primary and secondary prevention efforts. Therefore, as a secondary aim, we sought to examine if key demographic or clinical factors moderated the effects of Aβ and tau on cognition. We hypothesized that stronger associations between Aβ and tau with cognition would be observed in the presence of key ADRD risk factors, including older age, apolipoprotein E (APOE) ε4 carrier status, and lower education.^22^

## 2 METHODS

### 2.1 Participants

The FHS is a longitudinal observational cohort that began community‐based recruitment of the Original Cohort in 1948 followed by the enrollment of the Second Generation Cohort (children of the Original Cohort and their spouses) in 1971 and enrollment of the Third Generation Cohort (grandchildren of the Original Cohort and children of the Offspring Cohort) in 2002.^20^ Prospective, longitudinal surveillance of the Second and Third Generation Cohorts have occurred every four years. Beginning in 2015, individuals enrolled in the Second and Third Generation Cohorts were invited to participate in a PET imaging substudy with data collection occurring between September 2015 to June 2022. Eligibility for participation in the PET imaging substudy included (1) Age 30 years or older, (2) Completion of a prior FHS MRI; and (3) Absence of significant neurological conditions including dementia and clinical stroke. As previously described,^23^ FHS participants undergo continual dementia and stroke surveillance using a combination of cognitive screening and comprehensive monitoring. Of the 5,124 Second Generation Cohort participants, 3,171 attended the most recent examination with 68 undergoing PET imaging and, of those, 67 completing cognitive assessments. Of the 4,095 Third Generation Cohort participants, 2,430 have completed the most recent examination with 356 undergoing both PET imaging and cognitive assessments. The study sample for the current analyses included 414 individuals who underwent Aβ PET imaging. Due to funding availability, a subset of 322 individuals completed tau PET imaging. The time interval between cognitive testing and PET imaging ranged from 0 to 1.5 years with a mean of 0.2 ± 0.2 years.

The study sample was primarily comprised of individuals identifying as non‐Hispanic White as participants were descendants or kin‐by‐marriage of members of the Original Cohort recruited from Framingham, Massachusetts, beginning in 1948. The cohort is not representative of the current population demographic as racial and ethnic diversity in the community has increased over time (U.S. Census).

### 2.2 Assessments

Participants completed cognitive assessments, which were administered by trained research assistants using standardized administration procedures.^24^ The current analyses include assessments of (1). Verbal Memory (Weschler Memory Scale [WMS] Logical Memory [LM] Immediate and Delayed Recall), (2). Visual Memory (WMS Visual Reproductions Delayed Recall), (3). Attention/Executive Function (Trail Making Test Part B [Trails B] time to completion, Digit Span Backwards), (4). Language (Boston Naming Test [BNT]−30 item version), and 5). Visuospatial (Hooper Visual Organization Test [HVOT]). For domains that included more than one cognitive test, the test results were added together to create a composite score. Trails B and HVOT were naturally log‐transformed to normalize their distributions and the logged Trails B scores were multiplied by −1 so higher scores indicated better performances across all tests and domains. Scores from all domains were converted to z‐scores based on the study sample's mean and standard deviation. Additionally, a global cognition score (PC1) was derived from the first unrotated factor of a principal components analysis (PCA) that included WMS LM, VR, and Paired Associates Immediate and Delayed Recall, Trails B, Similarities, and HVOT.^25^

**RESEARCH IN CONTEXT**

1. **Systematic review**: Relevant literature was reviewed using databases such as PubMed. While cerebral amyloid beta and tau have been associated with cognitive decline and dementia in late life, less is known about the relationship in midlife, a period that may be important for prevention efforts.
2. **Interpretation**: Cerebral amyloid and tau were not cross‐sectionally associated with cognition overall, yet our study suggests that younger adults and apolipoprotein E ε4 carriers be more vulnerable to cognitive changes in the context of early amyloid beta and tau accumulation.
3. **Future directions**: Future studies should (a) examine the longitudinal associations between cerebral amyloid beta and tau with cognition at midlife, and (b) examine a broader array of factors that may shape risk and resilience to the cognitive effects associated with early amyloid beta and tau accumulation at midlife.

### 2.3 Aβ and tau PET imaging

As previously described,^26^, ^27^, ^28^ cerebral Aβ PET imaging was conducted using an injection of 8.5‐15 mCi ^11^C‐Pittsburgh compound B (PiB) followed by a 60‐minute dynamic 3D acquisition and reconstruction in 39 frames (8 × 15 s, 4 × 60 s, and 27 × 120 s). Tau PET was performed following a 10.0 ± 1.0 mCi ^18^F‐flortaucipir (FTP) bolus injection with images collected over 80–100 min using 4  5 min frames. PiB and FTP PET imaging were conducted on the same day. Structural T1‐weighted images (6800 msec; echo time, 3.1 msec; flip angle, 9°; voxel size, 0.98 × 0.98 × 1.2 mm) were obtained using a Philips Achieva 3T scanner. T1 images were collected with a mean of 0.2 ± 0.2 years from PET imaging. T1 images were processed using FreeSurfer v6.0 (http://surfer.nmr.mgh.harvard.edu/). Cortical and subcortical regions of interest (ROIs) were derived using the FreeSurfer Desikan‐Killiany atlas (https://surfer.nmr.mgh.harvard.edu/fswiki/CorticalParcellation) and aseg atlas (https://surfer.nmr.mgh.harvard.edu/ftp/articles/fischl02‐labeling.pdf), respectively. Mean PET images were coregistered with the FreeSurfer‐processed T1 images with a 6° of freedom rigid body registration using SPM version 12 (http://www.fil.ion.ucl.ac.uk/spm). PET images were collected using two different cameras over the study period, a 5‐Ring GE Discovery MI and a Siemens ECAT HR+. PiB and FTP data collected using the GE camera were smoothed using a 6 mm Gaussian filter for harmonization. PiB distribution volume ratio (DVR) and FTP standardized uptake value ratio (SUVR) were derived using the bilateral cerebellum gray matter as the reference region. Partial volume correction was not performed given the relatively young age of the sample and minimal atrophy present. A global PiB composite, termed frontal, lateral, and retrosplenial (FLR), was computed as the weighted average of inferior temporal, middle temporal, superior temporal, banks, transverse temporal, supramarginal, inferior parietal, superior parietal, insula, lateral orbitofrontal, pars orbitalis, pars triangularis, pars opercularis, caudal middle frontal, rostral middle frontal, superior frontal, medial orbitofrontal, rostral anterior cingulate, caudal anterior cingulate, posterior cingulate, precuneus, and isthmus cingulate regions. The composite was then log‐transformed for normality. Centiloids (CL) were also derived from the coregistered PiB PET images following Klunk et al. guidelines.^29^ For FTP images, five regions were interrogated based on their susceptibility to early AD pathology, the entorhinal cortex, rhinal cortex, inferior temporal lobe, fusiform gyrus, and middle temporal lobe.^30^, ^31^ All PET variables reflect the nonweighted averages of the right and left hemispheres.

### 2.4 Covariates and effect modifiers

Covariates included age (years), age squared (years^2^), sex (male or female), education (less than college graduate or college graduate or higher), PET camera (GE or Siemens), and the time interval between PET and cognitive data collection (years). For exploring effect modifiers, age was dichotomized using a median split of <55 years or ≥55 years, apolipoprotein (APOE) carrier status was dichotomized as no ε4 alleles or ≥one copy of the ε4 allele, and education was dichotomized as <college graduate or ≥college graduate.

### 2.5 Statistical analysis

The sample was characterized using descriptive statistics. Associations between Aβ and tau PET retention with cognition were evaluated using linear regression models adjusting for age, age squared, sex, education, PET camera, and the time interval between PET and cognitive data collection. Effect modification by age group, APOE ε4 status, and education were examined by adding an interaction term with the PET region to the linear regression models. Interaction results with a raw *p*‐value < 0.05 were further examined with stratified analyses. All statistical tests were two‐sided. Raw *p*‐values are presented in the manuscript and tables with significance after false discovery rate (FDR correction (*p* < 0.05) indicated. Analyses were conducted using SAS version 9.4.

## 3 RESULTS

### 3.1 Participants characteristics

A total of 423 participants (mean age 57 ± 10 years, 50% female) were included in the sample (Tables 1 and 2). Most of the sample (94%) had Aβ PET levels less than 20 CLs. Supplemental Figure 1 displays associations between global Aβ PET and entorhinal tau PET binding.

**TABLE 1:** Demographic and clinical characteristics of the sample.

Parameter	All *N* = 423
Age, years	57 ± 10
Female, n (%)	213 (50%)
Race and ethnicity, n (%) Non‐Hispanic White Unknown	421 (>99%) 2 (<1%)
Education, n (%) No college degree College degree or higher	148 (35%) 275 (65%)
APOE ε4 allele, n (%)	95 (23%)
Interval between cognitive testing and PET Imaging, years	0.2 ± 0.2
Verbal memory^a^	(*N* = 413) 0.00 ± 1.00
Visual memory^a^	(*N* = 419) 0.00 ± 1.00
Attention/executive^a^	(*N* = 406) 0.00 ± 1.00
Language^a^	(*N* = 394) 0.00 ± 1.00
Visuospatial^a^	(*N* = 419) 0.00 ± 1.00
Global cognition^a^	(*N* = 397) 0.00 ± 1.00
Aβ Centiloid, n (%) <20 20‐39 ≥40	(*N* = 403) 380 (94%) 14 (4%) 9 (2%)
Global Aβ, DVR	(*N* = 414) 1.08 ± 0.11
Entorhinal tau, SUVR	(*N* = 322) 1.05 ± 0.09
Rhinal tau, SUVR	(*N* = 317) 1.10 ± 0.10
Inferior temporal tau, SUVR	(*N* = 322) 1.14 ± 0.08
Middle temporal tau, SUVR	(*N* = 322) 1.09 ± 0.08
Fusiform tau, SUVR	(*N* = 322) 1.14 ± 0.08

*Note*: Values represent mean ± standard deviation unless otherwise noted.

Abbreviations: Aβ, amyloid beta, APOE, apolipoprotein E, DVR, distribution volume ratio, PET, positron emission tomography, SUVR, standardized uptake value ratio.

^a^ Values standardized to z‐scores.

**TABLE 2:** Demographic and clinical characteristics of the sample with stratification based on age, APOE ε4, and education group.

Parameter	Age < 55 *N* = 169	Age ≥55 *N* = 254	APOE ε4 *N* = 95	Non‐APOE ε4 *N* = 315	>College degree *N* = 275	≥College degree *N* = 148
Age, years	48 ± 5	64 ± 7	57 ± 10	57 ± 10	56 ± 10	59 ± 9
Female, n (%)	87 (52%)	126 (50%)	48 (51%)	160 (51%)	142 (52%)	71 (48%)
Race and ethnicity, n (%) Non‐Hispanic White Unknown	169 (100%) 0 (0%)	252 (99%) 2 (1%)	95 (100%) 0 (0%)	313 (99%) 2 (1%)	273 (99%) 2 (1%)	148 (100%) 0 (%)
Education, n (%) No college degree College degree or higher	46 (27%) 123 (73%)	102 (40%) 152 (60%)	27 (28%) 68 (72%)	115 (36%) 200 (64%)	275 (100%) 0 (0%)	0 (0%) 148 (100%)
APOE ε4 allele, n (%)	42 (26%)	53 (22%)	95 (100%)	0 (0%)	68 (25%)	27 (19%)
Interval between cognitive testing and PET Imaging, years	0.2 ± 0.2	0.2 ± 0.2	0.2 ± 0.2	0.2 ± 0.2	0.2 ± 0.3	0.2 ± 0.1
Verbal memory^a^	0.17 ± 0.99	−0.11 ± 0.99	−0.06 ± 1.04	0.00 ± 0.98	0.19 ± 0.96	−0.35 ± 0.98
Visual memory^a^	0.23 ± 0.92	−0.15 ± 1.02	0.01 ± 1.09	0.00 ± 0.97	0.15 ± 0.98	−0.28 ± 0.98
Attention/Executive^a^	0.29 ± 0.92	−0.20 ± 1.01	−0.14 ± 1.02	0.04 ± 0.98	0.16 ± 0.93	−0.31 ± 1.06
Language^a^	0.01 ± 1.00	0.00 ± 1.00	0.11 ± 1.02	−0.02 ± 0.99	0.16 ± 0.98	−0.31 ± 0.96
Visuospatial^a^	0.24 ± 0.98	−0.16 ± 0.98	−0.04 ± 1.03	0.00 ± 0.97	0.04 ± 0.98	−0.08 ± 1.04
Global cognitio^a^	0.29 ± 0.93	−0.19 ± 1.00	−0.01 ± 1.05	−0.01 ± 0.98	0.27 ± 0.89	−0.50 ± 1.00
Aβ Centiloid, n (%) <20 20‐39 ≥40	(*N* = 169) 165 (98%) 0 (0%) 4 (2%)	(*N* = 234) 215 (92%) 14 (6%) 5 (2%)	(*N* = 87) 77 (89%) 8 (9%) 2 (2%)	(*N* = 303) 292 (97%) 4 (1%) 7 (2%)	(*N* = 260) 250 (96%) 6 (2%) 4 (2%)	(*N* = 143) 130 (91%) 8 (6%) 5 (3%)
Global Aβ, DVR	1.04 ± 0.04	1.11 ± 0.13	1.11 ± 0.12	1.07 ± 0.11	1.08 ± 0.12	1.08 ± 0.09
Entorhinal tau, SUVR	1.05 ± 0.07	1.06 ± 0.11	1.08 ± 0.11	1.05 ± 0.09	1.06 ± 0.09	1.04 ± 0.11
Rhinal tau, SUVR	1.08 ± 0.08	1.11 ± 0.12	1.12 ± 0.12	1.09 ± 0.10	1.05 ± 0.09	1.08 ± 0.12
Inferior temporal tau, SUVR	1.12 ± 0.06	1.16 ± 0.09	1.15 ± 0.09	1.14 ± 0.08	1.14 ± 0.08	1.13 ± 0.08
Middle temporal tau, SUVR	1.07 ± 0.07	1.12 ± 0.09	1.11 ± .09	1.09 ± 0.08	1.10 ± 0.08	1.09 ± 0.08
Fusiform tau, SUVR	1.13 ± 0.06	1.15 ± 0.08	1.15 ± 0.09	1.13 ± 0.07	1.14 ± 0.07	1.13 ± 0.09

*Note*: Values represent mean ± standard deviation unless otherwise noted.

Abbreviations: Aβ, amyloid beta, APOE, apolipoprotein E, DVR, distribution volume ratio, PET, positron emission tomography, SUVR, standardized uptake value ratio.

^a^ Values standardized to z‐scores.

### 3.2 Aβ, tau, and cognition

There was an association of higher entorhinal and inferior temporal tau with poorer global cognition, but the results did not survive multiple comparisons correction (Table 3). In the overall sample, no other associations were identified.

**TABLE 3:** Associations of Aβ and tau with cognition.

Parameter	Global Cognition β (95% CI), *p*‐value	Verbal Memory β (95% CI), *p*‐value	Visual Memory β (95% CI), *p*‐value	Attention/ Executive β (95% CI), *p*‐value	Language β (95% CI), *p*‐value	Visuospatial β (95% CI), *p*‐value
Global Aβ	0.01 (−0.09, 0.10), *p* = 0.91	−0.03 (−0.13, 0.07), *p* = 0.50	0.04 (−0.07, 0.14), *p* = 0.47	0.03 (−0.07, 0.13), *p* = 0.51	0.03 (−0.08, 0.13), *p* = 0.59	0.03 (−0.07, 0.14), *p* = 0.55
Entorhinal tau	−0.13 (−0.23, −0.03), *p* = 0.01	−0.06 (−0.16, 0.04), *p* = 0.23	−0.05 (−0.15, 0.06), *p* = 0.38	−0.11 (−0.21, 0.00), *p* = 0.05	−0.04 (−0.15, 0.07), *p* = 0.46	−0.10 (−0.21, 0.01), *p* = 0.09
Rhinal tau	−0.10 (−0.20, 0.01), *p* = 0.07	−0.07 (−0.17, 0.03), *p* = 0.18	−0.04 (−0.15, 0.07), *p* = 0.43	−0.07 (−0.18, 0.04), *p* = 0.20	−0.04 (−0.15, 0.08), *p* = 0.52	−0.09 (−0.21, 0.02), *p* = 0.10
Inferior temporal tau	−0.12 (−0.23, −0.02), *p* = 0.03	−0.07 (−0.17, 0.04), *p* = 0.20	−0.07 (−0.18, 0.05), *p* = 0.24	−0.07 (−0.19, 0.04), *p* = 0.22	−0.06 (−0.17, 0.06), *p* = 0.35	−0.13 (−0.24, −0.01), *p* = 0.03
Middle temporal tau	−0.08 (−0.20, 0.04), *p* = 0.17	−0.03 (−0.15, 0.08), *p* = 0.58	−0.07 (−0.20, 0.05), *p* = 0.25	−0.01 (−0.13, 0.12), *p* = 0.91	−0.05 (−0.18, 0.07), *p* = 0.41	−0.11 (−0.24, 0.02), *p* = 0.09
Fusiform tau	−0.08 (−0.19, 0.02), *p* = 0.12	−0.05 (−0.15, 0.05), *p* = 0.35	−0.02 (−0.13, 0.09), *p* = 0.68	−0.07 (−0.18, 0.04), *p* = 0.24	−0.01 (−0.13, 0.10), *p* = 0.82	−0.08 (−0.19, 0.03), *p* = 0.18

*Note*: β (SE) and uncorrected *p*‐values derived from linear regression models examining the associations between Aβ and tau PET retention with cognition adjusting for age, age squared, sex, education, PET camera, and interval between cognitive testing and PET imaging. No results survived multiple correction (FDR‐corrected *p*‐value < 0.05).

Abbreviations: Aβ, amyloid beta; CI, confidence interval; FDR, false discovery rate; PET, positron emission tomography.

### 3.3 The effect of age on the associations of Aβ and Tau with cognition

**FIGURE 1:**
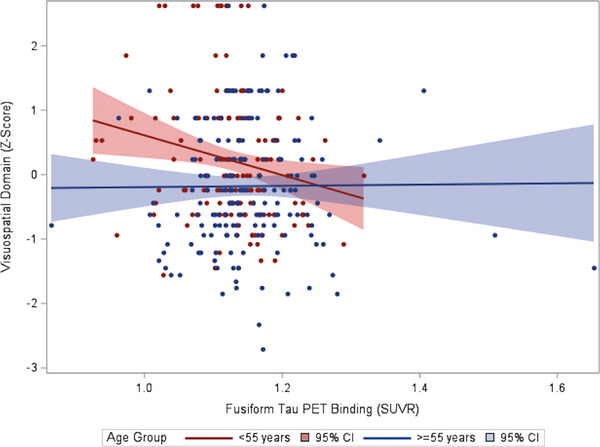
Scatterplot of the association between fusiform tau positron emission tomography (PET) binding and visuospatial functioning by age group.

We observed an interaction between age group and fusiform gyrus tau in their association with visuospatial function (*p* = 0.02). As seen in Figure 1, the age <55 group demonstrated poorer visuospatial functioning in the presence of higher fusiform tau relative to the ≥55 age group. Stratified analyses further indicated a significant association between higher fusiform gyrus tau and poorer visuospatial function in the age <55 group (*n* = 147, beta = −0.27, 95% CI −0.46 to −0.07, *p* = 0.007), which survived FDR‐correction. The association did not meet statistical significance in the ≥55 age group (*n* = 175, beta = 0.01, 95% CI −0.13 to 0.15, *p* = 0.90). We did not observe any other interactions with age group.

### 3.4 The effect of APOE on the associations of Aβ and tau with cognition

**FIGURE 2 alz14060-fig-0002:**
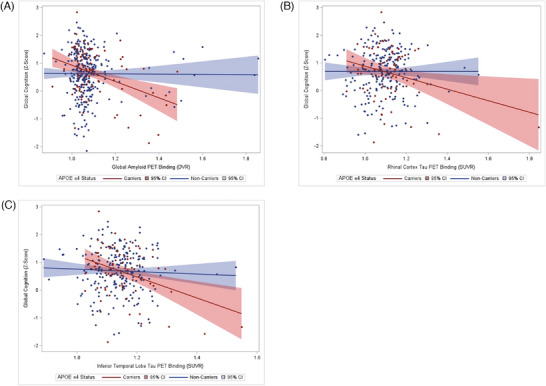
Scatterplot of the associations between global amyloid beta (Aβ) (A), rhinal tau (B), and inferior temporal lobe tau (C) positron emission tomography (PET) binding with global cognition by apolipoprotein ε4 status

As displayed in Table 4, APOE ε4 carrier status moderated associations between PET imaging and cognition across multiple cognitive domains. APOE ε4 interacted with global Aβ in relation to global cognition, visual memory, and language. There were significant interactions between APOE ε4 and entorhinal tau for verbal memory and language. APOE ε4 interacted with rhinal tau in relation to global cognition, verbal memory, and language. Significant interactions between APOE ε4 and inferior temporal lobe tau were observed for global cognition and language. There was also an interaction between APOE ε4 and fusiform gyrus tau for language. Across outcomes, APOE ε4 carriers demonstrated poorer cognition in the presence of higher PET binding relative to nonε4 carriers (Figure 2A‐C). None of the stratified results survived FDR correction.

**TABLE 4 alz14060-tbl-0004:** Interactions and stratified analyses of the associations between Aβ and tau with cognition by APOE ε4 carrier status

Global Aβ*APOE (*p*‐value)	Stratified analyses β (95% CI), *p*‐value
Global cognition (*p* = 0.006)	ε4: −0.21 (−0.44, 0.02), *p* = 0.08
	non‐ε4: 0.09 (−0.03, 0.20), *p* = 0.13
Verbal memory (*p* > 0.05)	
Visual memory (*p* = 0.008)	ε4: −0.14 (−0.39, 0.10), *p* = 0.25
	non‐ε4: 0.04 (−0.07, 0.14), *p* = 0.47
Attention/Executive (*p* > 0.05)	
Language (*p* = 0.02)	ε4: −0.16 (−0.42, 0.10), *p* = 0.22
	non‐ε4: 0.09 (−0.03, 0.21), *p* = 0.16
Visuospatial (*p* > 0.05)	

Entorhinal tau*APOE (*p*‐value)	**Stratified Analyses β (95% CI), *p*‐value**
Global cognition (*p* > 0.05)	
Verbal memory (*p* = 0.04)	ε4: −0.16 (−0.34, 0.02), *p* = 0.09
	non‐ε4: 0.00 (−0.12, 0.12), *p* = 0.94
Visual memory (*p* > 0.05)	
Attention/Executive (*p* > 0.05)	
Language (*p* = 0.02)	ε4: −0.18 (−0.38, 0.02), *p* = 0.08
	non‐ε4: 0.03 (−0.11, 0.16), *p* = 0.69
Visuospatial (*p* > 0.05)	

Rhinal tau*APOE (*p*‐value)	**Stratified Analyses β (95% CI), *p*‐value**
Global cognition (*p* = 0.03)	ε4: −0.20 (−0.39, −0.01), *p* = 0.04
	non‐ε4: −0.04 (−0.17, 0.09), *p* = 0.56
Verbal memory (*p* = 0.02)	ε4: −0.19 (−0.38, −0.01), *p* = 0.04
	non‐ε4: 0.00 (−0.12, 0.12), *p* = 0.98
Visual memory (*p* > 0.05)	
Attention/Executive (*p* > 0.05)	
Language (*p* = 0.02)	ε4: −0.16 (−0.38, 0.05), *p* = 0.13
	non‐ε4: 0.04 (−0.10, 0.18), *p* = 0.55
Visuospatial (*p* > 0.05)	

Inferior temporal tau*APOE (*p*‐value)	Stratified Analyses β (95% CI), *p*‐value
Global cognition (*p* = 0.02)	ε4: −0.21 (−0.43, 0.01), *p* = 0.06
	non‐ε4: −0.06 (−0.18, 0.07), *p* = 0.36
Verbal memory (*p* > 0.05)	
Visual memory (*p* > 0.05)	
Attention/Executive (*p* > 0.05)	
Language (*p* = 0.02)	ε4: −0.25 (−0.49, −0.01), *p* = 0.04
	non‐ε4: 0.02 (−0.11, 0.16), *p* = 0.72
Visuospatial (*p* > 0.05)	

Middle temporal tau*APOE (*p*‐value)	**Stratified Analyses β (95% CI), *p*‐value**
Global cognition (*p* > 0.05)	
Verbal memory (*p* > 0.05)	
Visual memory (*p* > 0.05)	
Attention/Executive (*p* > 0.05)	
Language (*p* > 0.05)	
Visuospatial (*p* > 0.05)	

Fusiform tau*APOE (*p*‐value)	**Stratified Analyses β (95% CI), *p*‐value**
Global cognition (*p* > 0.05)	
Verbal memory (*p* > 0.05)	
Visual memory (*p* > 0.05)	
Attention/Executive (*p* > 0.05)	
Language (*p* = 0.04)	ε4: −0.13 (−0.35, 0.10), *p* = 0.27
	non‐ε4: 0.05 (−0.09, 0.18), *p* = 0.51
Visuospatial (*p* > 0.05)	

*Note*: Uncorrected *p*‐values for the interaction between cognition and APOE ε4 carrier status for Aβ and tau PET retention derived from linear regression models adjusting for age, age squared, sex, education, PET camera, interval between cognitive testing and PET imaging, cognition, and APOE ε4 carrier status. Stratified analyses by APOE ε4 carrier status were performed when the interaction term had a raw *p*‐value < 0.5. β (SE) and *p*‐values for the stratified analyses are derived from linear regression models adjusting for age, age squared, sex, education, PET camera, and interval between cognitive testing and PET imaging. No results survived multiple correction (FDR‐corrected *p*‐value < 0.05).

Abbreviations: Aβ = amyloid beta, APOE = apolipoprotein E, CI, confidence interval; FDR, false discovery rate; PET = positron emission tomography.

### 3.5 The effect of education on the associations of Aβ and tau with cognition

An interaction with education group was observed for the association between global Aβ and verbal memory (*p* = 0.009) with the ≥college degree group displaying poorer cognition in the presence of higher Aβ relative to the <college degree group. However, in stratified analyses, the association between global Aβ and verbal memory was not significant in either group (<college degree (*n* = 140, beta = 0.20, 95% CI −0.02 to 0.41, *p* = 0.07; ≥college degree (*n* = 264, beta = −0.10, 95% CI −0.22 to 0.01, *p* = 0.08). No other significant interactions with education group were observed.

## 4 DISCUSSION

The continuous range of Aβ and tau accumulation at midlife was not associated with cognition in our cross‐sectional community‐based cohort. However, the findings do not suggest that early amyloid beta and tau accumulation are completely benign as certain subgroups demonstrated cognitive vulnerability with higher Aβ and regional tau accumulation. Specifically, higher fusiform gyrus tau was associated with poorer visuospatial function in adults <55 years old. In addition, APOE ε4 carrier status interacted with Aβ and tau PET binding to worsen cognition across multiple domains. These findings suggest that even early accumulations in Aβ and tau may have a pernicious effect on cognition among APOE ε4 carriers and younger adults, suggesting a potential role for earlier interventions in subgroups at elevated risk.

Overall, Aβ and tau PET retention were not cross‐sectionally associated with cognition in the sample at large. Few studies have examined the impact of Aβ and tau PET retention at midlife. In the WRAP cohort, elevations in Aβ and tau positivity assessed using PiB and ^18^F‐MK‐6240 PET imaging were not retrospectively associated with cognition at the time of enrollment, when the sample had a mean age of 54 years.^19^ However, individuals classified as Aβ and tau PET positive displayed an approximate three‐fold faster decline in global cognition when followed longitudinally over an average of 8 years. Our study has important differences as we utilized a cross‐sectional design in a community‐based cohort without AD enrichment. In addition, rather than dichotomizing, we chose to explore the full range of Aβ and tau accumulation. Most of our cohort had low levels of Aβ, which may be contributing to the null associations with cognition in the overall sample. A study of older adults without cognitive impairment conducted across multiple cohorts indicated that global Aβ PET retention values between 15 and 18.5 CL were the lowest thresholds at which future cognitive was detected.^32^ While tau PET retention more robustly correlates with cognitive decline than amyloid PET^8^, the exact threshold and degree of dependence on Aβ remains unclear in asymptomatic adults. In a sample of cognitively unimpaired older adults, Hanseeuw et al.^6^ reported that longitudinal increases in neocortical tau were associated with cognitive decline over a mean of eight years, which were contingent upon elevations in baseline Aβ. In contrast, other studies in cognitively intact older adults have identified elevations in tau that occur in the absence of Aβ positivity and are associated with poorer cognition.^9^, ^10^ Thus, longitudinal studies beginning at middle age or earlier will be critical for providing further clarity on the temporal trajectories of Aβ and tau accumulation and their associations with cognition in individuals who will and will not ultimately develop clinical AD.

While we did not observe any main effects of cognition with Aβ or tau PET retention, our study identified significant effect modifiers. Given that Aβ and entorhinal tau generally increase with age^18^ and the fact that age is the most significant risk factor for AD,^33^ we hypothesized that age would strengthen the association between Aβ and tau PET retention with cognition. In contrast, higher fusiform gyrus tau was associated with poorer visuospatial function only in the younger age group. While the results were unexpected, they may indicate higher cognitive vulnerability in individuals who develop higher neuropathological protein accumulation earlier in life. A study comparing individuals with early and late‐onset AD reported that early onset was associated with higher amyloid and tau PET burden at similar disease stages.^34^ Prior work from the FHS Cohort has demonstrated that hypertension is associated with more advanced brain aging at midlife relative to late‐life,^35^ similarly suggesting that insults at younger ages may have a larger impact on brain health. In addition, with advancing age, broader neuropathological burden, including TDP‐43, alpha‐synuclein, and white matter pathology, increases.^36^ Therefore, the strength of association between tau and cognition may reduce in the context of additional pathological processes. Across the cognitive domains examined, only visuospatial functioning was associated with fusiform gyrus tau in the younger age group. Visuospatial dysfunction is one of the earliest cognitive symptoms in AD, emerging in the preclinical stage.^34^, ^37^, ^38^ The fusiform gyrus is integral to objection recognition and spatial localization.^37^ The visuospatial task used in this study, the HVOT, requires recognition of objects that have been fragmented^39^ and has been shown to activate the fusiform gyrus in functional MRI studies.^40^ Prior PET imaging studies have indicated that the fusiform gyrus is among the regions most susceptible to early AD pathology in cognitively unimpaired adults.^15^ Longitudinal studies will be necessary to evaluate if younger adults with higher fusiform gyrus tau are ultimately at increased risk of clinical progression.[Table alz14060-tbl-0004]

The APOE ε4 allele is the strongest known genetic susceptibility marker for AD^41^ and APOE isoforms are involved in Aβ oligomerization, aggregation, and removal.^42^ In a sample of cognitively unimpaired and MCI older adults, Weigand et al.^43^ reported a significant moderating effect of APOE ε4 status on the associations between memory and medial temporal tau PET retention with tau exerting a more deleterious effect on memory in ε4 carriers. Our study extends these findings by demonstrating poorer cognition among APOE ε4 carriers in the presence of higher Aβ and tau beginning at midlife. As most of our cohort was Aβ PET negative, the results suggest that the detrimental impact of Aβ and tau for cognition may manifest at subthreshold levels among individuals at elevated genetic risk for AD. In our study, stratified analyses by APOE ε4 carrier status were not significant. However, our study was likely underpowered to detect these associations as less than a quarter of our community‐based sample were APOE ε4 carriers.

Finally, we examined educational attainment as a potential effect modifier given that it is the best‐established proxy for cognitive reserve education group interacted with global Aβ in relation to verbal memory with those with a college degree or higher unexpectedly displaying poorer performance in the presence of higher Aβ relative to individuals with less than a college degree. However, in stratified analyses, the association between global Aβ and verbal memory was not significant in either education group. In prior studies, higher education has been found to attenuate the impact of neuropathological burden on cognition.^44^, ^45^, ^46^ These findings may not have been replicated in our study given the high level of educational attainment in the sample.

Our study has several limitations, which should be considered when interpreting the results. First, the study sample was overwhelmingly non‐Hispanic White and well‐educated and, thus, is not currently representative of the larger community from which they were recruited. Therefore, generalizability is limited, and future work is needed to evaluate how broader sociocultural factors may influence the impact of Aβ and tau on cognition. In addition, while our overall sample was fairly large, we may have been underpowered for stratified analyses, particularly for examinations of APOE ε4 as less than a quarter of the sample were ε4 carriers. Another limitation is the cross‐sectional study design. While no main effects were observed between cognition with Aβ and tau PET retention, it remains unclear whether there are associations between the these AD markers and longitudinal cognitive trajectories. Also, our study used FTP tau PET imaging, which has off‐binding effects^2^. While these effects tend to be of smaller magnitude at younger ages,^47^ tau tracers with higher binding affinity may demonstrate different effects.

Overall, our study suggests that early increases in Aβ and tau accumulation may worsen cognition in APOE ε4 carriers and in younger adults. Longitudinal studies will be important for evaluating if these associations influence cognitive trajectories and risk of future conversion to MCI and dementia. The results highlight the importance of community‐based studies for understanding the role of early Aβ and tau accumulation on cognition, as well as identifying critical timepoints for intervention among subgroups at elevated risk.[Fig alz14060-fig-0002]

## CONFLICT OF INTEREST STATEMENT

Dr. Gonzales has personal stock in AbbVie. Dr. Seshadri has consulted for Eisai and Biogen outside the current work. Dr. O'Donnell is an employee of Novartis unrelated to her role on this project. Dr. Ghosh, Ms. Thibault, Dr. Tanner, Dr. Satizabal, Dr. DeCarli, Dr. El Fakhri, Dr. Johnson, Dr. Beiser, and Dr. Pase report no disclosures. Author disclosures are available in the supporting information.

## CONSENT STATEMENT

The PET imaging substudy was approved by the Institutional Review Boards at Boston University Medical Center and the Massachusetts General Hospital. All participants provided written informed consent at the time of enrollment.

## Supporting information

Supporting Information

Supporting Information
