# Supplementary figures and images for: Associations of cerebral amyloid beta and tau with cognition from midlife
Source: Alzheimers Dement. 2024 Jul 22;20(9):5901–11. doi: 10.1002/alz.14060 (PMC11497641; doi:10.1002/alz.14060)

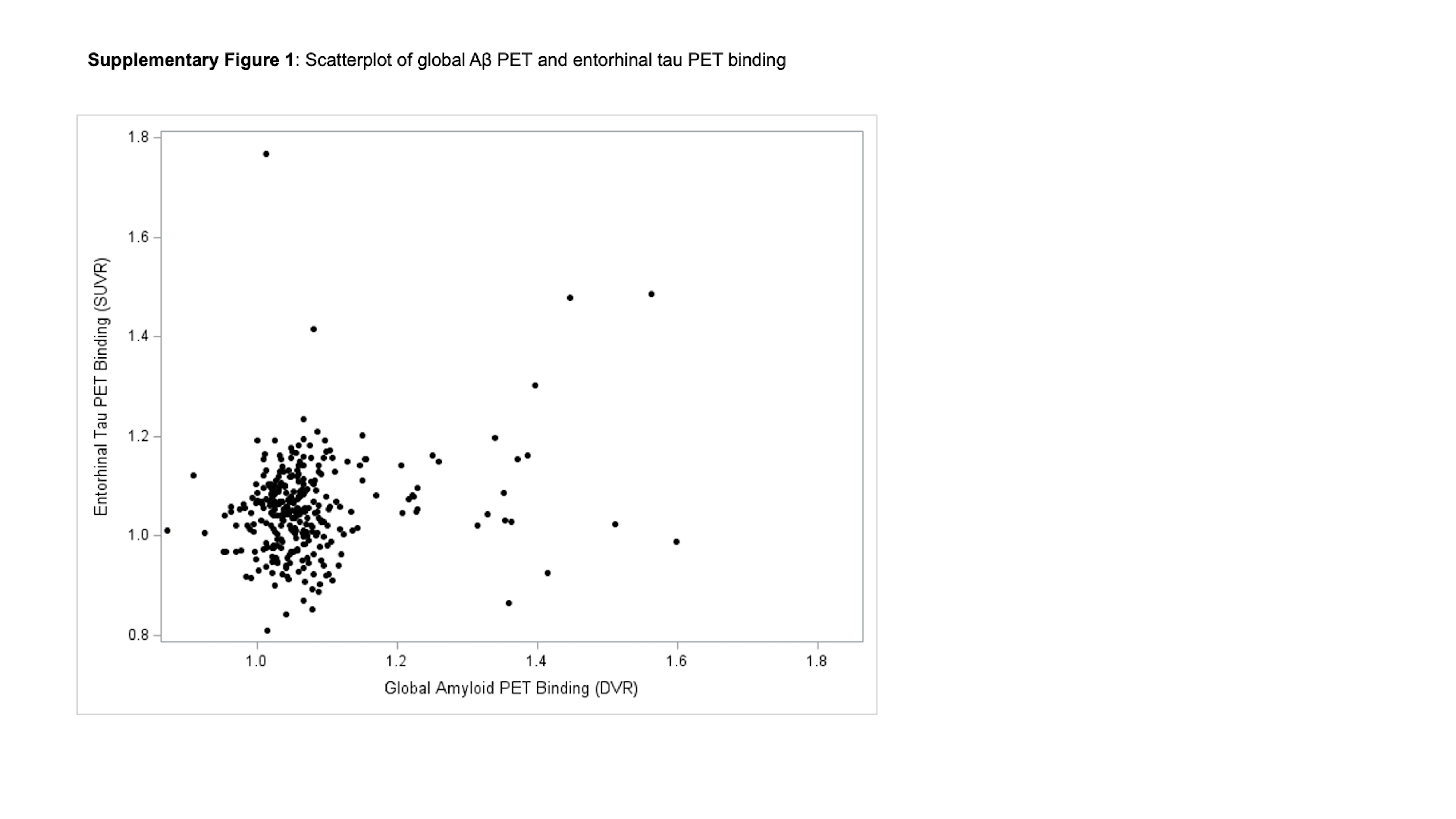

Supplement: Supplementary file 1 — Supporting Information [file ALZ-20-5901-s001.jpg]
